# Antithrombotic agent usage before ictus in aneurysmal subarachnoid hemorrhage: relation to hemorrhage severity, clinical course, and outcome

**DOI:** 10.1007/s00701-023-05556-z

**Published:** 2023-03-14

**Authors:** Hanna Kultanen, Anders Lewén, Elisabeth Ronne-Engström, Per Enblad, Teodor Svedung Wettervik

**Affiliations:** Department of Medical Sciences, Section of Neurosurgery, Uppsala University, SE-751 85 Uppsala, Sweden

**Keywords:** Aneurysmal subarachnoid hemorrhage, Anticoagulant, Antiplatelet, Clinical outcome, Hijdra score, Neurointensive care

## Abstract

**Background:**

The number of patients with aneurysmal subarachnoid hemorrhage (aSAH) who are on antithrombotic agents before ictus is rising. However, their effect on early brain injury and disease development remains unclear. The primary aim of this study was to determine if antithrombotic agents (antiplatelets and anticoagulants) were associated with a worse initial hemorrhage severity, rebleeding rate, clinical course, and functional recovery after aSAH.

**Methods:**

In this observational study, those 888 patients with aSAH, treated at the neurosurgical department, Uppsala University Hospital, between 2008 and 2018 were included. Demographic, clinical, radiological (Fisher and Hijdra score), and outcome (Extended Glasgow Outcome Scale one year post-ictus) variables were assessed.

**Results:**

Out of 888 aSAH patients, 14% were treated with antithrombotic agents before ictus. Seventy-five percent of these were on single therapy of antiplatelets, 23% on single therapy of anticoagulants, and 3% on a combination of antithrombotic agents. Those with antithrombotic agents pre-ictus were significantly older and exhibited more co-morbidities and a worse coagulation status according to lab tests. Antithrombotic agents, both as one group and as subtypes (antiplatelets and anticoagulants), were not associated with hemorrhage severity (Hijdra score/Fisher) nor rebleeding rate. The clinical course did not differ in terms of delayed ischemic neurological deficits or last-tier treatment with thiopental and decompressive craniectomy. These patients experienced a higher mortality and lower rate of favorable outcome in univariate analyses, but this did not hold true in multiple logistic regression analyses after adjustment for age and co-morbidities.

**Conclusions:**

After adjustment for age and co-morbidities, antithrombotic agents before aSAH ictus were not associated with worse hemorrhage severity, rebleeding rate, clinical course, or long-term functional recovery.

**Supplementary information:**

The online version contains supplementary material available at 10.1007/s00701-023-05556-z.

## Introduction

There is a rise in patients being treated with antithrombotic agents, including antiplatelets and anticoagulants, as the general population gets older and to a greater extent exhibits cardiovascular and thromboembolic diseases [1]. In parallel, there is a growing concern for adverse effects from these agents if a hemorrhage should occur, especially in the brain [12, 33]. Aneurysmal subarachnoid hemorrhage (aSAH) is a severe form of hemorrhagic stroke with a high burden of mortality and morbidity [24]. Functional outcome depends to a great extent on the severity of the initial hemorrhage and if rebleeding occurs [24, 39, 42]. Therefore, there is an interest in the effect of antithrombotic agents on aSAH incidence, severity, clinical course, and long-term outcome.

Previous studies have focused on whether antithrombotic agents increase the risk of aSAH and if it increase case fatality if bleeding should occur. Aspirin has received most attention. On one hand, some studies indicate that aspirin decreases the risk of aneurysmal development, growth, and rupture due to its anti-inflammatory effects [6, 14, 15, 28, 29], although other studies rather indicate an increased risk for aSAH due to induced coagulopathy [13, 23]. Less is known about other antiplatelets (e.g., clopidogrel), but they have more consistently been associated with a higher aSAH risk [13, 23]. There is limited knowledge about the aSAH course for patients on antiplatelets, but most studies indicate that the initial injury severity, the risks of complications, case fatality, and functional outcome are not worse in this group [4, 7, 13, 37]. It is also worth noting that as more patients are treated with endovascular aneurysm occlusion and thereafter require antiplatelets as a thromboembolic prophylaxis, emerging studies indicate that antiplatelets may exert a protective effect on delayed ischemic neurological deficits and infarctions in the acute stage after aSAH [20]. Regarding anticoagulants, most studies have focused on vitamin K antagonists (VKAs), and epidemiological studies indicate that long-term use is associated with an increased risk of developing aSAH [23, 26], but not consistently [13]. The number of studies on aSAH cases with anticoagulant treatment before ictus is limited. Initial studies on VKA use before ictus indicated very poor functional recovery in the case of aSAH [25], whereas more modern studies have not indicated worse outcomes [5, 7]. In addition, the role of novel oral anticoagulants (NOACs) has increased in usage in recent years, but their effect on the risk of aSAH and outcome in case of hemorrhage remains elusive.

Altogether, antithrombotic agents in aSAH patients constitute an emerging clinical challenge, but it is still today not fully clear what the net effects of different types of antithrombotic agents are on the risk of developing aSAH and its clinical course and how these agents should be managed in case of aneurysmal rupture. In this study, we aimed to study if patients on antithrombotic agents before ictus exhibited a worse hemorrhage severity (amount of blood and rebleeding), clinical course, and long-term functional outcome after aSAH.

## Materials and methods

### Patients

Patients with aSAH admitted to the Department of Neurosurgery at the University Hospital in Uppsala, Sweden, 2008–2018, were eligible for this study. Out of 910 adult patients with aSAH aged 18 years and older, 22 had been initially treated at another NIC and were excluded. Consequently, the study population was 888 aSAH patients.

### Treatment protocol

Patients with aSAH were admitted to our neurointensive or neurointermediate care unit and were managed in accordance with our standardized treatment protocol [27, 31, 32, 34, 35, 40]. Treatment goals were ICP ≤ 20 mm Hg, CPP ≥ 60 mm Hg, systolic blood pressure ≥ 100 mm Hg, pO_2_ ≥ 12 kPa, arterial glucose 5–10 mmol/L (mM), electrolytes within normal ranges, slight hypervolemia with 0 fluid balance after aneurysm occlusion, and body temperature < 38 °C. Unconscious aSAH patients were intubated and mechanically ventilated. Aneurysms were occluded as early as possible, by endovascular embolization or surgical clipping. An external ventricular drain (EVD) was preferentially inserted for ICP monitoring and possibly cerebrospinal fluid drainage in patients who were unconscious and/or with acute hydrocephalus. Nimodipine was administered to all patients after admission to our department. Delayed ischemic neurological deficits (DIND) were defined as a new onset of focal neurological deficit or deterioration in consciousness, not explained by, e.g., hydrocephalus, rebleeding, or meningitis. If there was no manifestation of cerebral infarction on computed tomography (CT), a HHH (hypertension, hypervolemia, and hemodilution) therapy was initiated [9]. Thiopental infusion and decompressive craniectomy (DC) were last-tier treatments for intracranial hypertension.

For patients who were on any antithrombotic agent before ictus, the specific agent was immediately withdrawn following aSAH. Antiplatelets were generally only withdrawn, whereas vitamin K antagonists were also reversed with prothrombin complex concentrate and vitamin K. NOAC was withdrawn and sometimes treated with a single dose of tranexamic acid and/or prothrombin complex concentrate, although the specific NOAC type, dabigatran, was treated with withdrawal and administration of the antidote idaruzicumab. Tranexamic acid was not administered before aneurysm occlusion on a general basis [22].

### Clinical and radiological variables

Clinical variables, including demography, co-morbidities, admission variables, and treatments, were extracted from medical records. The extent of co-morbidities was evaluated according to the Charlson co-morbidity index [30], which is a numeric summary measure of co-existing diseases. Blood and coagulation lab tests including hemoglobin, platelets, international normalized ratio (INR), and activated partial thromboplastin time (aPTT) at admission were assessed. The tests were conducted at the accredited laboratory of the Department of Clinical Chemistry at Uppsala University Hospital. The Hijdra score was assessed as a radiological variable of hemorrhage severity [16, 18]. The Hijdra sum score (max score = 42) is the sum of the cistern and ventricle scores. The cistern score is based on radiological grading on the amount of blood (grades 0–3) in ten cisterns (max score = 30) and the ventricle score on the amount of blood (grades 0–3) in the four ventricles (max score = 12). These assessments were conducted by one of the authors (TSW). The Fisher grade was also assessed [10] on the initial CT scans. Rebleeding was defined as the combination of clinical deterioration and more SAH on the follow-up CT.

### Outcome

Functional outcome was evaluated according to the Extended Glasgow Outcome Scale (GOS-E) 12 months after ictus [36, 41], by a trained personnel using structured telephone interviews. GOS-E has eight categories of outcome, from death (1) to upper good recovery (8). Functional outcome was dichotomized as favorable/unfavorable (GOS-E 5–8/1–4).

### Statistical analyses

Categorical data were reported as numbers (proportion) and ordinal/continuous data as medians (interquartile range (IQR)). Differences in demography, admission variables, hemorrhage severity, clinical course, treatments, and functional outcome between those with antithrombotic agents before ictus and those without were assessed with chi-square analysis or Mann–Whitney *U* test, depending on the type of data. A multiple linear regression analysis was conducted, and variables that are thought to be related to hemorrhage severity, including age, sex, the Charlson co-morbidity index, aneurysm location and size, days from symptoms to CT, and antithrombotic agents, were included as independent variables with the Hijdra sum score as the dependent variable. A similar regression was conducted, in which antithrombotic agents were replaced by the subgroups, antiplatelets and anticoagulants, before ictus. In addition, multiple logistic regression analyses were conducted with mortality and favorable outcome as dependent variables, respectively, and antithrombotic agents together with the baseline pre-ictal variables, age and the Charlson co-morbidity index, as independent variables. A *p*-value < 0.05 was considered statistically significant. Missing values were rare, and these cases were excluded from the analyses, i.e., no imputation was conducted. The statistical analyses were conducted using SPSS version 28 (IBM Corp., Armonk, NY, USA).

## Results

### Patient characteristics

In the entire aSAH group of 888 patients (Table 1), median age was 59 (IQR 51–67) years, most patients were females (67%), and the Charlson co-morbidity index was in median 0 (IQR 0–0). At admission, WFNS was in median 2 (IQR 1–4), 10% exhibited an abnormal (unreactive) pupillary response, and the aSAH was diagnosed in median on day 0 (IQR 0–1) post-ictus. The Fisher grade was in median 3 (IQR 3–4), and the median Hijdra sum score was 17 (IQR 9–25). Most aneurysms were located in the anterior circulation (84%), the median aneurysm diameter was 6 mm (IQR 4–8), and the majority were treated with endovascular interventions (72%). Sixty percent received some type of antithrombotic agent (LMWH and/or antiplatelet) after aneurysm occlusion. More than half (56%) received an EVD, 20% developed DIND, 7% were treated with thiopental, and 6% with DC. Seventeen percent of the cases were deceased, whereas 48% had recovered favorably at 1 year post-ictus.

**Table 1:** Demography, admission status, treatments, and outcome for all patients, patients with antithrombotic agents, and patients without antithrombotic agents. *p*-value for significant difference between the groups

	All	Antithrombotic agents	No antithrombotic agents	*p*-value
Patients *n* (%)	888	125 (14)	763 (86)	NA
Age, median (IQR)	59 (51–67)	68 (61–74)	58 (50–66)	**0.001**
Sex (female/male), *n* (%)	597/291 (67/33%)	70/55 (56/44%)	527/236 (69/31%)	**0.004**
Charlson co-morbidity index, median (IQR)	0 (0–0)	1 (0–1)	0 (0–0)	**0.001**
WFNS grade, median (IQR)	2 (1–4)	2 (1–4)	2 (1–4)	0.70
Pupillary status (abnormal), *n* (%)	89 (10%)	12 (10%)	77 (10%)	0.87
Time from onset to CT (days), median (IQR)	0 (0–1)	0 (0–0)	0 (0–1)	0.37
Fisher, median (IQR)	3 (3–4)	3 (3–4)	3 (3––4)	0.79
Hijdra ventricle score, median (IQR)	1 (0–4)	2 (0–4)	1 (0–3)	0.07
Hijdra cistern score, median (IQR)	15 (7–23)	16 (8–23)	15 (7–23)	0.75
Hijdra score, median (IQR)	17 (9–25)	17 (10–25)	17 (9–25)	0.61
Aneurysm anterior/posterior, *n* (%)	748/140 (84/16%)	101/24 (81/19%)	647/116 (85/15%)	0.26
Aneurysm size (mm), median (IQR)	6 (4–8)	6 (4–9)	6 (4–8)	0.19
Rebleeding, *n* (%)	71 (8%)	7 (6%)	64 (8%)	0.29
ICP monitor (none/EVD/Codman/both), *n* (%)	337/499/14/38 (38/56/2/4%)	42/79/2/2 (34/63/2/2%)	295/420/12/36 (39/55/2/5%)	0.22
Treatment (no/embolization/clipping/both), *n* (%)	62/637/182/7 (7/72/21/1%)	9/96/20/0 (7/77/16/0%)	53/541/162/7 (7/71/21/1%)	0.38
Antiplatelets after embolization (none/LMWH/ASA/DAPT), *n* (%)	353/122/356/57 (40/14/40/6%)	45/16/55/9 (36/13/44/7%)	308/106/301/48 (40/14/39/6)	0.78
Hematoma evacuation, *n* (%)	99 (11%)	9 (7%)	90 (12%)	0.13
DIND, *n* (%)	169 (20%)	27 (22%)	142 (19%)	0.43
Thiopental, *n* (%)	59 (7%)	5 (4%)	54 (7%)	0.20
Decompressive craniectomy, *n* (%)	49 (6%)	3 (2%)	46 (6%)	0.10
NIC days, mean (IQR)	13 (9–18)	13 (9–18)	13 (10–18)	0.71
GOS-E, median (IQR)	3 (4–6)	3 (1–5)	5 (3–6)	**0.001**
Favorable/unfavorable outcome, *n* (%)	411/447 (48/52%)	30/89 (25/75%)	381/358 (52/48%)	**0.001**
Mortality, *n* (%)	149 (17%)	31 (26%)	118 (16%)	**0.007**

*ASA* aspirin, *CT* computed tomography, *DAPT* double antiplatelet treatment, *DIND* delayed ischemic neurologic deficit, *EVD* external ventricular drain, *GOS-E* Glasgow Outcome Scale Extended, *ICP* intracranial pressure, *IQR* interquartile range, *LMWH* low molecular weight heparin, *NIC* neurointensive care, *WFNS* World Federation of Neurosurgical Societies

Those which are bold were statistically significant (*p* < 0.05)

### Antithrombotic agents—frequency, type, indication, and management after ictus

**Fig. 1:**
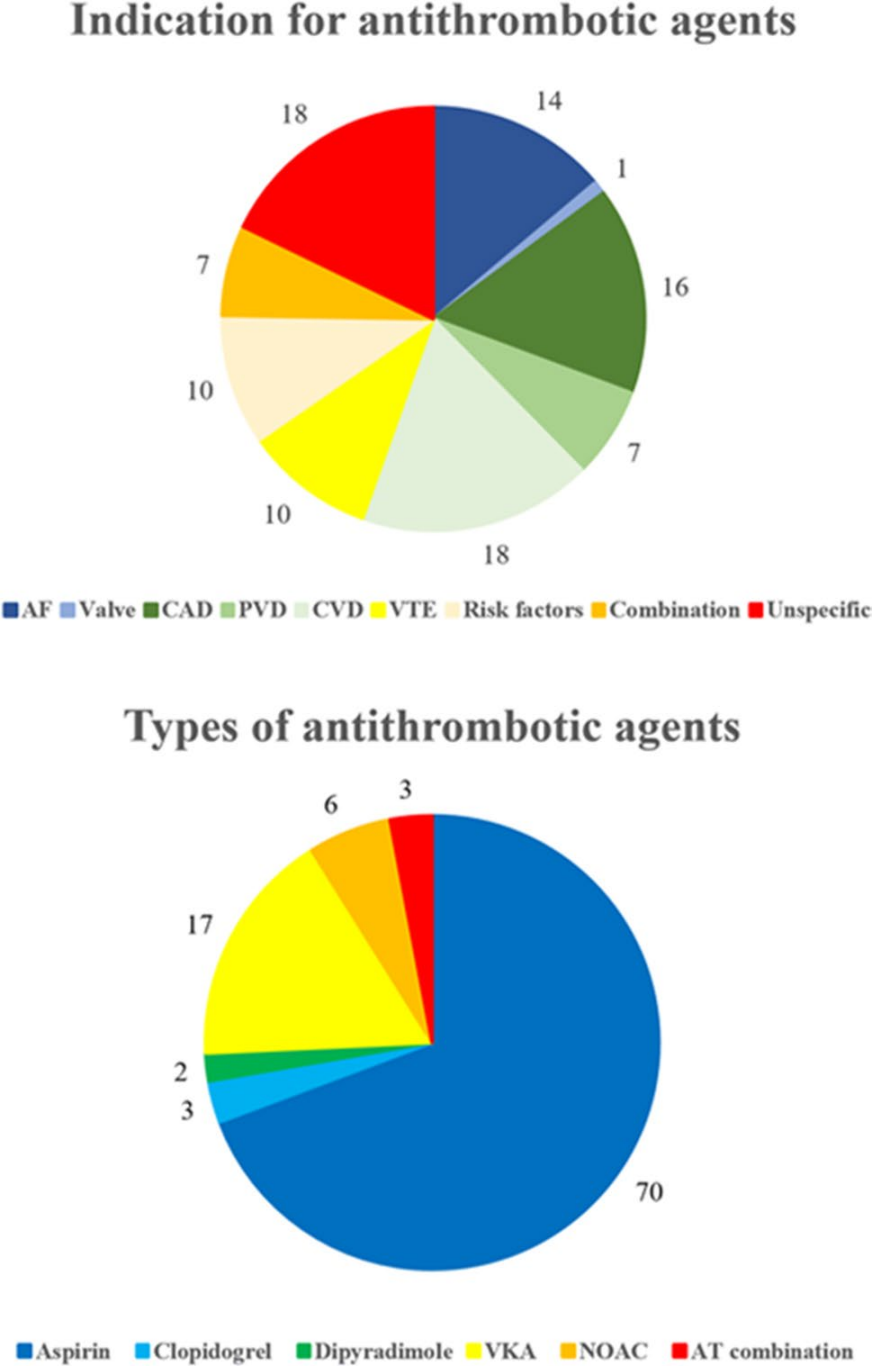
The figures demonstrate the distribution (percentage) of indications for treatment with antithrombotic agents and the types of antithrombotic agents these patients had before ictus. AF, atrial fibrillation; AT, antithrombotic; CAD, coronary artery disease; CVD, cerebrovascular disease; PVD, peripheral vascular disease; VKA, vitamin K antagonist; VTE, venous thromboembolism

As demonstrated in Table 2 and Fig. 1, 125 (14%) patients were on antithrombotic agents before ictus. The majority of them were treated with single-therapy antiplatelets (74%), almost exclusively with aspirin (70%), whereas the remainder were on anticoagulants (22%) or a combination of antithrombotic agents (3%). The main indications for antithrombotic agents before ictus were previous coronary artery disease (16%), atrial fibrillation (14%), cerebrovascular disease (10%), or venous thromboembolism (10%) (Fig. 1). Most patients with antiplatelets were managed with immediate withdrawal alone (Table 2), whereas those with anticoagulants typically received some type of reversal treatment (prothrombin complex concentrate and vitamin K).

**Table 2:** Management of antithrombotic agents

Types	Agents	Patients, *n*	Reversal	Patients, *n* (%)
Antiplatelets	Aspirin	87	Withdrawal	74 (85%)
			Withdrawal + desmopressin	1 (1%)
			Unknown	12 (14%)
	Clopidogrel	4	Withdrawal	4 (100%)
	Dipyridamole	2	Withdrawal	2 (100%)
Anticoagulants	Vitamin K antagonist	21	Withdrawal	5 (24%)
			PCC + vitamin K	15 (71%)
			PCC + vitamin K + desmopressin	1 (5%)
	NOAC	7	Withdrawal	3 (43%)
			Tranexamic acid + PCC	1 (14%)
			PCC	2 (29%)
			Tranexamic acid + PCC + Praxbind	1 (14%)
Antithrombotic combination	Dual antiplatelets	2	Withdrawal	1 (50%)
			Withdrawal + platelet transfusion + desmopressin	1 (50%)
	Dual antiplatelets + LMWH	1	Platelet transfusion + desmopressin	1 (100%)
	Aspirin + LMWH	1	Withdrawal	1 (100%)

*LMWH* low molecular weight heparin, *PCC* prothrombin complex concentration

### Antithrombotic agents—relation to demography, admission status, and clinical course

Patients with antithrombotic agents were older (*p* < 0.001) and more often male (*p* = 0.004) and exhibited more co-morbidities with a higher a Charlson co-morbidity index (*p* < 0.001), lower thrombocytes (*p* = 0.005), and higher INR at admission (*p* < 0.001; Table 1 and Supplementary Table 1). There was no difference in WFNS grade, extent of pupillary abnormalities, aneurysm location or size, development of DIND, the extent and type of ICP monitoring, or the degree of last-tier ICP treatment with thiopental and DC between the groups (Table 1).

### Antithrombotic agents—relation initial hemorrhage severity and rebleeding

**Fig. 2:**
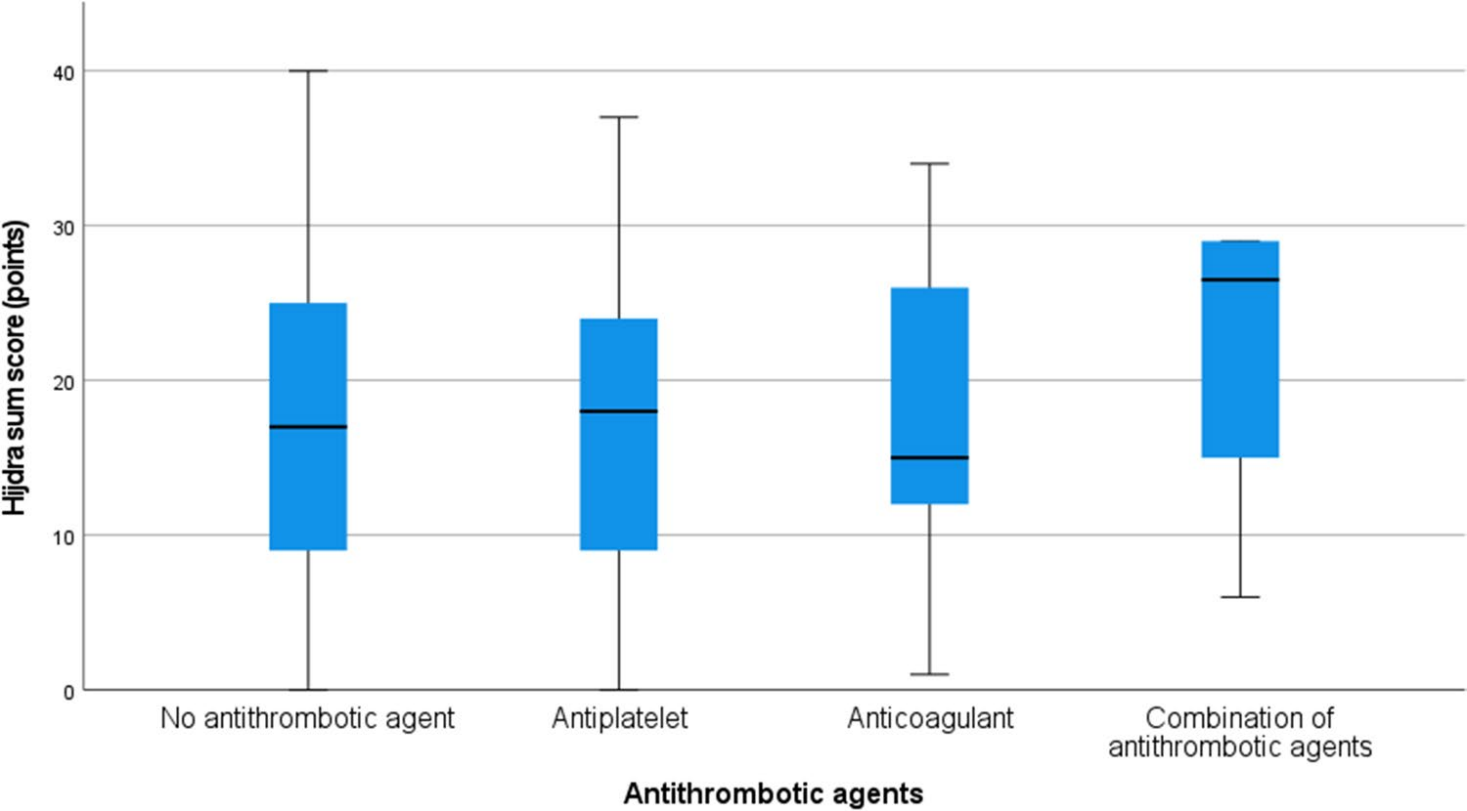
Antithrombotic agent vs Hijdra score (median and IQR). Non-AT, no antithrombotic agents; AP, antiplatelet; AK, anticoagulant. The boxplot shows the median and interquartile range of Hijdra sum score for patients without antithrombotic agents before ictus as well as those with antiplatelets, anticoagulants, and a combination of different antithrombotic agents before ictus. There was no significant difference between the groups, but a clear trend of increased Hijdra score in patients with a combination of antithrombotic agents

In univariate analyses, there was no difference in radiological variables (Fisher grade, Hijdra ventricle score, Hijdra cistern score, or Hijdra sum score) or in rebleeding rate between those with or without antithrombotic agents before ictus (Table 1). Consistently, antithrombotic agents were not independently associated with the Hijdra sum score in a multiple linear regression analysis (Table 3). Higher age (*β* = 0.19, *p* < 0.001) and shorter duration between the onset of symptoms and CT imaging (*β* =  − 0.31, *p* < 0.001) were independently associated with a higher Hijdra sum score (Table 3 and Fig. 2). In a similar regression, when antithrombotic agents were replaced by the subgroups antiplatelets and anticoagulants, these separate subgroups were also not associated with the Hijdra sum score (Table 3).

**Table 3:** Prediction of hemorrhage severity according to the Hijdra score—a multiple regression analysis

Regression 1		
Prediction of the Hijdra sum score; antithrombotic agents—the entire group		
Variables	*β*	*p*-value
Age	0.19	**0.001**
Sex (female)	− 0.01	0.70
Charlson co-morbidity index	0.04	0.24
Aneurysm location (posterior)	0.06	0.07
Aneurysm size	0.04	0.20
Days from symptom to first CT	− 0.31	**0.001**
Antithrombotic agent (yes)	− 0.03	0.40
Regression 2		
Prediction of the Hijdra sum score; antithrombotic agents—different types		
Variables	*β*	*p*-value
Age	0.18	**0.001**
Sex	− 0.01	0.72
Charlson co-morbidity index	0.04	0.29
Anterior/posterior aneurysm	0.06	0.07
Aneurysm size	0.04	0.20
Days from symptom to first CT	− 0.31	**0.001**
Antithrombotic agent		
Antiplatelet	− 0.02	0.60
Anticoagulant	− 0.01	0.87

Regression 1, ANOVA *p* < 0.001, *R*^2^ = 0.14

Regression 2, ANOVA *p* < 0.001, *R*^2^ = 0.14

Those which are bold were statistically significant (*p* < 0.05)

### Antithrombotic agents—relation to mortality and neurological recovery 1 year post-ictus

Patients with antithrombotic agents exhibited an increased mortality (26 vs 16%, *p* = 0.007) and were less prone to reach a favorable functional recovery (25 vs 52%, *p* < 0.001) at 1 year post-ictus (Table 1). These associations were not statistically significant in multiple logistic regression analyses for mortality and favorable outcome (Table 4), after adjustment for age and the Charlson co-morbidity index. Higher age and the Charlson co-morbidity index were independently associated with a higher mortality and lower rate of favorable outcome in these regressions (Table 4).

**Table 4:** Antithrombotic agents in relation to mortality and favorable outcome—a multiple logistic regression analysis

Mortality				
Variables	Regression 1		Regression 2	
	OR (95% CI)	*p*-value	OR (95% CI)	*p*-value
Age	1.04 (1.02–1.05)	**0.001**	1.04 (1.02–1.05)	**0.001**
Charlson co-morbidity index	1.38 (1.07–1.77)	**0.01**	1.38 (1.07–1.79)	**0.01**
Antithrombotic agent (yes)	1.05 (0.63–1.78)	0.85	NA	NA
Antiplatelet (yes)	NA	NA	1.00 (0.56–1.79)	1.00
Anticoagulant (yes)	NA	NA	1.47 (0.61–3.50)	0.39
Favorable outcome				
Variables	Regression 3		Regression 4	
	OR (95% CI)	*p*-value	OR (95% CI)	*p*-value
Age	0.96 (0.94–0.97)	**0.001**	0.96 (0.94–0.97)	**0.001**
Charlson co-morbidity index	0.52 (0.37–0.74)	**0.001**	0.52 (0.36–0.74)	**0.001**
Antithrombotic agent (yes)	0.69 (0.42–1.13)	0.14	NA	NA
Antiplatelet (yes)	NA	NA	0.78 (0.45–1.38)	0.39
Anticoagulant (yes)	NA	NA	0.50 (0.20–1.26)	0.14

*CI* confidence interval, *OR* odds ratio

Those which are bold were statistically significant (*p* < 0.05)

## Discussion

In this study including 888 aSAH patients, 14% were medicated with antithrombotic agents before ictus, which in most cases included single therapy with aspirin. The patients with antithrombotic agents were generally older and with a higher rate of co-morbidities. Despite their drug-induced coagulopathy, they did not exhibit a worse initial hemorrhage severity (Hijdra score) or higher frequency of rebleeding nor required hematoma evacuation more often. They did experience a higher mortality and lower rate of favorable outcome, but this was explained by their higher age and co-morbidities, according to multiple regression analyses. These main observations require some reflection.

First, the observed percentage of patients with antithrombotic agents (14–10% with antiplatelets, 3% with anticoagulants, and 1% with a combination of antithrombotic agents) is consistent with previous studies [4, 5, 37]. As expected, these patients were older and exhibited more co-morbidities.

Second, being medicated with antithrombotic agents before ictus was associated with a more deranged coagulation, including lower thrombocytes and higher INR. Still, antithrombotic (both as one group and subtypes) agents were not associated with a worse initial hemorrhage severity or a higher rate of rebleeding. This is to our knowledge the first study on this matter that has conducted such a granular analysis of hemorrhage burden by using the Hijdra score. However, our findings were consistent with other studies not finding any association between aspirin or VKA with the more crude hemorrhage scale “Fisher grade” [4, 5]. One important explanation for the lack of association could be both poor drug compliance and also the lack of drug effect from, e.g., aspirin resistance [8]. In addition, although patients with antiplatelet- (except aspirin) and anticoagulant-induced coagulopathy may be more prone to develop aSAH on an epidemiological level [13, 23, 26], this does not necessarily imply that these cases also fare worse. For those additional aSAH cases attributed to coagulopathy from pre-ictal antithrombotic agents that would not have otherwise occurred, it could be speculated if their aneurysms were different in a way that more rapidly terminated the bleeding and led to a smaller hemorrhage burden [19]. In addition, we did not see any association between antithrombotic agents and rebleeding rate, which was in line with some previous studies on aspirin and VKA [4, 5, 37, 38]. From a theoretic point of view, it appears surprising that drug-induced coagulopathy does not translate into more rebleedings, but it is possible that modern management including immediate withdrawal and possibly reversal of the antithrombotic agents, vigilant blood pressure management, and early aneurysm occlusion minimize this risk.

Third, those with antithrombotic agents did not exhibit a worse clinical status at admission (WFNS grade and pupillary status), and they did not experience a worse neurointensive care course including DIND, more EVD monitoring and CSF drainage, or more treatment with thiopental and DC. These findings indicate that antithrombotic agents did not necessarily influence the acute clinical course in a negative way, which is consistent with previous studies [4, 5, 37].

Fourth, although our patients with antithrombotic agents showed a higher mortality and lower rate of favorable outcome in univariate analyses, multiple logistic regression analyses indicated that this was explained by their higher age and their co-morbidities. More historic case series have indicated very poor outcome for those with anticoagulants [25]. However, our findings corroborate recent studies on antiplatelets [4, 7, 13, 37] and anticoagulants [5, 7, 13] in aSAH, although the literature is scarce.

Fifth, hemostatic management in aSAH patients with antithrombotic agents before ictus remains controversial. Most of our patients on antiplatelets were managed with withdrawal alone. Further options included platelet transfusion and desmopressin. Routine use of platelet transfusion does not seem warranted as it has been independently associated with worse outcome in aSAH [21], and a randomized controlled trial on platelet transfusion for patients on antiplatelets with intracerebral hemorrhage showed that they fared worse with transfusion [2]. On the contrary, desmopressin seems more promising as it improves thrombocyte function and has been shown to reduce the rebleeding rate in aSAH [11], but more prospective studies are needed to validate this. Almost all patients on anticoagulants were treated with immediate VKA reversal, by using prothrombin complex concentrate and vitamin K, which likely immediately reduced any potentially increased rebleeding risk. The literature on VKA reversal in aSAH is limited, but a smaller case series indicated that prothrombin complex concentrate and vitamin K are effective to improve the coagulation status as well as outcome [3]. NOACs were uncommon in this study, and our management was rather heterogeneous. The literature is also limited in this area for aSAH, and it is likely that more specific antidotes for the various NOAC subtypes will be used in the near future. Furthermore, tranexamic acid in aSAH before aneurysm occlusion has received interest and controversy. Initial studies showed a lower rebleeding rate with tranexamic acid [17], but not in more recent studies and without any benefit on functional outcome [22]. However, it remains to be determined if aSAH patients with certain antithrombotic agents before ictus could be one subgroup of patients that benefits from such treatment.

### Methodological considerations

The literature is still relatively scarce of detailed studies on the course of aSAH patients treated with antithrombotic agents before ictus. This study was relatively large, and it provides the most detailed description of the association between antithrombotic agents and hemorrhage severity, by using the granular Hijdra score.

There are also some limitations. First, it has been estimated that around 10% of the aSAH do not survive to reach the emergency department [15]. Yet another percentage of the entire aSAH patients might have been in a too poor condition to benefit from neurointensive care and therefore not admitted to our unit. These two subgroups suffered from more severe initial hemorrhages, and we cannot exclude that these scenarios were more common for patients with antithrombotic agents before ictus. Second, the number of patients with VKA and particularly those with NOAC was small (*n* < 30), which decreased the reliability of the analyses of these cases. This is also the reason why we abstained from further subanalyses of specific antithrombotic agents. Third, management (continuation/withdrawal/reversal) of aspirin after aSAH diagnosis was not thoroughly documented in 12 patients; however, we anticipate that this reflected that they were withdrawn, and no reversal was given in accordance with our management policy. Fourth, although severe rebleedings are easy to recognize in the clinical setting, we cannot exclude the small rebleedings that were not detected nor reported. Fifth, we cannot exclude that some cases were treated with antithrombotic agents before ictus, and some co-morbidities were not recognized as this was a retrospective study.

## Conclusions

Although aSAH patients treated with antithrombotic agents before ictus were more coagulopathic, they did not exhibit a worse hemorrhage severity, higher rebleeding rate, or a worse clinical course during NIC. They generally showed a worse neurological recovery at 1 year, but this was rather explained by high age and more co-morbidities.

## Supplementary information

Below is the link to the electronic supplementary material.

Supplementary file1 (DOCX 12 KB)

## Data Availability

Data is available upon reasonable request.
