# Supplementary material for: Antithrombotic agent usage before ictus in aneurysmal subarachnoid hemorrhage: relation to hemorrhage severity, clinical course, and outcome
Source: Acta Neurochir (Wien). 2023 Mar 14;165(5):1241–50. doi: 10.1007/s00701-023-05556-z (PMC10140004; doi:10.1007/s00701-023-05556-z)
Supplement: Supplementary file 1 — Supplementary file1 (DOCX 12 KB) [file 701_2023_5556_MOESM1_ESM.docx]

**Supplementary table 1. Coagulation tests at admission**

| Variables | *All* | *Antithrombotic agents* | *No antithrombotic agents* | *p-value* |
| --- | --- | --- | --- | --- |
| Hemoglobin (g/dL), median (IQR) | 135 (126-144) | 134 (124-143) | 135 (126-144) | 0.56 |
| Thrombocytes (10^9^/unit), median (IQR) | 254 (212-304) | ***240 (191-294)*** | ***257 (215-307)*** | 0.005 |
| INR (ratio), median (IQR) | 1.0 (1.0-1.1) | ***1.1 (1.0-1.2)*** | ***1.0 (1.0-1.1)*** | 0.001 |
| aPTT(s), median (IQR) | 32 (30-35) | 33 (30-36) | 32 (30-34) | 0.053 |

aPTT = Activated partial thromboplastin time. INR = International normalized range. IQR = Interquartile range.
